# Spatially and Temporally Explicit Metagenomes and Metagenome-Assembled Genomes from the Comau Fjord (42°S), Patagonia

**DOI:** 10.1128/mra.00059-23

**Published:** 2023-05-15

**Authors:** Eduardo Castro-Nallar, Valentín Berríos-Farías, Beatriz Díez, Sergio Guajardo-Leiva

**Affiliations:** a Departamento de Microbiología, Facultad de Ciencias de la Salud, Universidad de Talca, Talca, Chile; b Centro de Ecología Integrativa, Universidad de Talca, Talca, Chile; c Department of Molecular Genetics and Microbiology, Pontificia Universidad Católica de Chile, Santiago, Chile; d Center for Climate and Resilience Research (CR)^2^, Santiago, Chile; e Millennium Institute Center for Genome Regulation (CGR), Santiago, Chile; University of Southern California

## Abstract

Microbes play an important role in coastal and estuarine waters. We present 93 metagenomes and 677 metagenome-assembled genomes (MAGs) from Comau Fjord, Patagonia (42°S), to further understand the microbial dynamics and their response to anthropogenic disturbances. These data represent a spatially (35-km transect) and temporally (2016 to 2019) explicit data set.

## ANNOUNCEMENT

Coastal and estuarine waters not only receive carbon and nutrients from rivers and other freshwater sources but also are a hot spot of disturbances of anthropogenic origin (1–5). Comau Fjord in northern Patagonia, Chile, is surrounded by national parks and privately protected land. However, the fjord is part of commercial and passenger transport routes, as well as open-cage aquaculture centers, which affect biodiversity by pouring hydrocarbons, antibiotics, and excess nutrients into the ecosystem (6–8). To date, no studies have addressed the extent and impact of these disturbances on the microbial communities of the Comau Fjord. The metagenomic data presented here will contribute to our understanding regarding the consequences of anthropogenic activities in the fjord.

We collected seawater over a period of 3 years at depths of 5 and 20 m, from the mouth to the end of the fjord (40 L per sample, using an electric water pump). The water was prefiltered using a 50-μm nylon mesh (Sefar) to avoid macroscopic organisms and debris, followed by filtering with 3-μm polycarbonate filters (Merck, Millipore) to collect eukaryotes and particle-attached prokaryotes. Free-living prokaryotes were collected in 0.22-μm polyethersulfone (PES) Sterivex filters (Merck, Millipore), and DNA from this fraction was obtained using phenol-chloroform extraction with the xanthogenate protocol (9–11). Sequencing libraries were constructed using Illumina TruSeq DNA kits and sequenced using Illumina instruments (see the Comau sample table at https://doi.org/10.6084/m9.figshare.21960866.v3).

We used default software parameters unless otherwise stated. We removed adapters (–detect_adapter_for_pe) and carried out filtering and trimming (-q 30 -l 100) using fastp 0.20.1 (12). We first estimated the genomic distances using Mash v2.3 (13) and checked whether the metagenomes formed clusters using the Hopkins statistic (>0.7). We then used agglomerative hierarchical clustering (the hclust function in the base R package), which resulted in 4 clusters (Table 1), as suggested by silhouette analysis (14). *De novo* metagenome coassembly was carried out on each of the four clusters using MEGAHIT v1.2.9 (–min-contig-len 1500) (15). To bin the resulting contigs (778,598), we used GroopM v2.0.0, CONCOCT v1.0.0, and MetaBAT v2.15 separately to then refine the bins using the binning refinement module in MetaWRAP v1.3 (-c 50 -x 10) (16–19). The metagenome-assembled genomes (MAGs) presented here are ≥50% complete and <10% contaminated (see MAG stats at https://doi.org/10.6084/m9.figshare.21960866.v3) (20). Taxonomic assignment was conducted using GTDB-tk 1.5.1 and database version R202 (21). Finally, to obtain the read abundances per MAG, we mapped the reads from each sample against the MAGs using CoverM v0.6.1 (–dereplicate –dereplication-ani 99 –min-read-aligned-percent 30 -m trimmed_mean) (total 517 dereplicated genomes) (https://github.com/wwood/CoverM).

**TABLE 1:** Assembly statistics for metagenomes and MAGs

Cluster	No. of contigs	Total length (bp)	Minimum length (bp)	Maximum length (bp)	Mean length (bp)	No. of refined MAGs
C1	140,400	666,764,307	1,500	95,119	4,749	128
C2	387,383	1,742,624,422	1,500	1,141,308	4,498	266
C3	138,518	666,137,931	1,500	427,687	4,809	160
C4	112,297	581,840,304	1,500	564,936	5,181	123

At the phylum level, the most abundant MAGs belonged to *Proteobacteria* (49%), *Bacteroidota* (31%), *Actinobacteriota* (7%), *Cyanobacteria* (4%), *Verrucomicrobiota* (2%), *Planctomycetota* (1.4%), and *Thermoplasmatota* (1.2%). Likewise, at the family level, the most abundant MAGs belonged to *Flavobacteriaceae* (26%), *Rhodobacteraceae* (20%), *Actinomarinaceae* (5%), *Cyanobiaceae* (4%), *Thioglobaceae* (4%), D2472 (3.7%), *Porticoccaceae* (3.2%), *Methylophilaceae* (2.8%), *Pseudohongiellaceae* (2.3%), *Schleiferiaceae* (2.2%), *Halieaceae* (1.8%), HTCC2089 (1.4%), *Akkermansiaceae* (1.3%), TMED25 (1%), and SAR86 (1%).

### Data availability.

This whole-metagenome shotgun project has been deposited at GenBank under the BioProject accession no. PRJNA729490. The version described in this paper is the first version.
